# Antifungal activity of curcumin nanoparticles and *Saccharomyces cerevisiae* against aflatoxigenic *Aspergillus flavus* isolated from broiler chickens rations in Egypt

**DOI:** 10.1186/s12866-026-04963-3

**Published:** 2026-04-29

**Authors:** Usama Hassan Abo-Shama, Eman Mahmoud El-Diasty, Doaa Ebrahim Mohamed, Reem Mohamed Alsaadawy, Haitham Helmy Sayed

**Affiliations:** 1 Department of Microbiology, Faculty of Veterinary Medicine, Sohag University, Sohag, 82524 Egypt; 2 Department of Mycology and Mycotoxins, Animal Health Research Institute, Agriculture Research Center, Giza, 12618 Egypt; 3 Department of Zoonoses, Faculty of Veterinary Medicine, Assiut University, Assiut, 71526 Egypt

**Keywords:** Aflatoxin B1, Aflatoxigenic, *Nor-1* gene, Seasonal variation, Total mold count (TMC), *Ver-1* gene

## Abstract

Fungi and mycotoxin contamination of feed poses a significant risk to broiler chickens (BC) and humans, as well as lowering feed quality. This study assessed fungal contamination of BC rations at Sohag Governorate, Egypt, throughout the year and evaluated aflatoxigenicity (AFTG) of *Aspergillus flavus* (*A. flavus*) isolates as well as antifungal activity (AA) of curcumin nanoparticles (Cur-NPs) and *Saccharomyces cerevisiae* (*S. cerevisiae*) against aflatoxigenic (AFT)* A. flavus* isolates. Mycological examination revealed that 92.9% of the investigated rations were contaminated by fungi, and total mould count ranged from 1 × 10 to 1.91 × 10^7^ CFU/g of ration. Isolated fungi belonged to 27 fungal genera, with predominance of genus *Aspergillus* followed by *Penicillium*, *Mucor*, *Cladosporium*, *Eurotium,* and *Scopulariopsis*. Furthermore,* Aspergillus* isolates were morphologically characterized into 20 different species, with predominance of *A. flavus* followed by *Aspergillus niger*, *Aspergillus terreus,* and *Aspergillus ustus,* respectively. Also, mycological examination revealed that fungal contamination of the rations was the highest in autumn, followed by summer, winter, and spring, respectively. Statistically, there were significant differences in the incidences of different fungal genera and *Aspergillus* spp. between the different year seasons (*P* < 0.001). Regarding AFTG of *A. flavus* isolates, 27.3% of them were AFT with variable degrees, and 90% of the strong AFT isolates were AFB1 producers, with high amounts ranging from less than 25 to 71 µg/L. Statistical analysis revealed the presence of a significant difference in the distribution of AFT *A. flavus* isolates through the different seasons (*P* < 0.001). PCR revealed that 100% and 30% of the investigated strong AFT *A. flavus* isolates (*n* = 10) harboured *nor*-1 and *ver*-1 genes, respectively. On the other hand, Cur-NPs exhibited strong AA against AFT *A. flavus* isolates, which increased with increasing Cur-NPs concentration. Also, the growth of all AFT *A. flavus* isolates was inhibited with *S. cerevisiae,* with an inhibition zone of 24.6 ± 0.64 mm^2^. Therefore, immediate essential control and preventive measures are needed to ensure food safety. Cur-NPs and *S. cerevisiae* are recommended for evaluation and use as effective fungicides to control AFT *A. flavus* and its potential for aflatoxin production in BC feeds.

**Supplementary Information:**

The online version contains supplementary material available at 10.1186/s12866-026-04963-3.

## Introduction

The Egyptian broiler chicken (BC) industry is a rapidly expanding sector [1] that plays a vital role in supplying affordable animal protein and supporting national food security [2]. However, as reported by Radwan et al*. *[3], the poultry industry faces numerous challenges, including both infectious and non-infectious diseases that represent major threats to production efficiency and profitability. Poultry are highly susceptible to mycoses and mycotoxicoses [4], and fungal diseases, which have become as significant as bacterial and viral infections, frequently leading to considerable morbidity and mortality [3]. Feed represents a critical route for disease transmission within farming systems [5] and serves as the principal source for the introduction of mycotoxins (MTs) into broiler flocks [6].

Moulds are ubiquitous in nature and frequently found in animal feeds along with their Mycotoxins (MTs) [6] causing a serious impact on human and animal health [7], as well as reduce the feed nutritive value [8], palatability, and feed spoilage [9]. According to El-Maghraby et al*.* [10] and Alkhursan et al*.* [11]. They can grow in feed ingredients and finished feeds, and produce MTs before or after harvest, while being transported, processed, stored, or fed. Temperature, humidity, and feed handling and processing all have a big impact on fungal growth and MT production during these phases [4].

Egypt lies in a tropical and subtropical arid climate [12] favour the growth of fungi and MTs contamination [13]. In addition, the Egyptian poultry industry depends mainly on imported feed ingredients, so there is a great opportunity for their contamination by moulds and MTs throughout the food system [14]. Otherwise, the major poultry feed ingredients in Egypt, corn and soybean, represent an excellent substrate for the growth of many fungi under the favorable conditions [4].

Some fungal genera, such as *Aspergillus*, *Fusarium*, *Mucor*, *Penicillium*, and *Rhizopus*, commonly contaminate poultry rations [15]. Occurrence of* Aspergillus* spp. in BC rations is of particular importance, where they are one of the most toxigenic fungi [4] and causative agents of feedstuffs spoilage [16]. In addition to that, aspergillosis caused by some *Aspergillus* spp. is an increasingly common fungal infection in birds and occasionally humans [4].

*Aspergillus flavus* (*A. flavus*) is the 5th most feared fungus [17]. It is a dominant fungus in poultry rations and a main producer of aflatoxins (AFs) [18]. AFs are one of the most toxicologically and economically significant MTs that pose the greatest potential risk to the health of humans and animals as feed contaminants [9], in addition to the reduction of the feed quality [19]. Among others, only 4 types of the identified AFs are commonly present in the feeds, namely aflatoxin B1 (AFB1), AFB2, AFG1, and AFG2 [20]. AFB1 is the most toxic and carcinogenic AF [8]. Therefore, it is important to investigate Aflatoxigenicity (AFTG) of *A. flavus* present in BC rations, especially for AFB1. In Egypt, the maximum permissible limits for AFs and AFB1 in feeds are 20 μg/Kg and 10 μg/Kg, respectively [21].

AFs cause various effects in poultry, including anorexia, decreased body weight gain, poor feed utilization, increased liver fat, and immunosuppression [22]. Additionally, hemorrhages, mutagenesis, teratogenesis, and carcinogenesis are associated with aflatoxicosis [23], and mortality is linked to acute aflatoxicosis [24]. Also, evidences indicate that AFs-induced immunosuppression causes many disease outbreaks and vaccination failures [25].

Contamination with AFs is one of the most dangerous food safety issues worldwide [26]. Poultry is the most consumed meat in Egypt [27], and BC represents 95% of its total consumption [28]. Several studies reported the presence of AFs residues in the meat of BC fed on AFs-contaminated feed [26]. Due to the high thermal stability of AFs, they aren't destroyed by processing and cooking, remain in the food, and are transferred to humans [29], posing a significant health hazard to consumers due to their mutagenic, carcinogenic, and teratogenic properties [20]**,** as well as occurrence of acute toxicity when they are consumed in high amounts [30]. AFs except AFM1 were classified as human carcinogens in group 1 by the International Agency for Research on Cancer [31] and are statistically linked with a high incidence of liver cancer in certain areas of South Africa, Egypt, and China [32].

It is mandatory to effectively control *A. flavus* [33] and AFs in poultry feeds for the protection of humans and birds, as well as ensuring the economic viability of the poultry industry [20]. Once AFs are produced, it is very difficult to eliminate them or reduce their contamination due to their high physical and chemical stability [19]. Furthermore, none of the present strategies to control AFs is sufficient to achieve the necessary safety and cost demands completely [20]. Therefore, the most effective way to avoid food contamination with AFs is to inhibit *A. flavus* growth in the foods [34].

Contamination with *A. flavus* can be prevented by the use of resistant varieties, application of good agricultural practices, and the use of chemical or biocontrol agents [35]. Modern agriculture has become increasingly dependent on the utilization of fungicides [36]. However, fungicide utilization poses various health risks and can result in environmental pollution in addition to its unfeasibility postharvest, so there is a need for the development of more eco-friendly methods to control *A. flavus* contamination that are free of the health risks associated with chemical fungicide utilization [37].

Nanotechnology has the potential to influence many aspects of food and agricultural systems, including food security [38]. Nanotechnology applications facilitate food preservation and nutrition enhancement. Nanoparticles are often used as food additives for food preservation and to protect from contamination [39]. Since nanoparticles have enhanced surface area and mass transfer rates, they seem to have greater penetrability, biological and chemical activity, enzymatic reactivity, and quantum characters in comparison to the large particles with the same composition [40]. Despite the inherent advantages and medical benefits of curcumin, its implementation in the food or pharmaceutical industry faces several challenges. Nanotechnology and nano-drug delivery systems may be the best options to overcome these challenges [41].

The biological control for fungal growth prevention and MTs production is considered one of the most promising, successful [42], and favored methods nowadays [34]. *Saccharomyces cerevisiae* (*S. cerevisiae*) is a safe, non-pathogenic yeast, able to grow on simple nutrients and colonize dry surfaces for a long time. Also, it doesn't produce allergenic spores, toxins, or antibiotics like the other microorganisms. *S. cerevisiae* has been extensively recommended to be used as a biocontrol agent in food due to its advantages, and its use instead of chemical preservatives could encourage the production of organic food free of chemical additives [34]. Furthermore, it has been highlighted as a probiotic in poultry farming, and its cell wall components have been shown to act as immunostimulants, growth promoters, and mycotoxin adsorbents [43].

Presence of moulds and MTs varies depending on the geographical location and the season of the year [11]. Determining the fungal contamination level of the rations and different contaminating fungi is a valuable indicator of the rations' hygienic quality and risk of MTs contamination and an essential prerequisite for the development of control or prevention strategies from fungal and MTs contamination for feed and food safety, as well as the avoidance of economic losses. Despite the growing global concern regarding fungal and AFs contamination in poultry feeds, significant gaps remain in Egypt, especially Upper Egypt. Revising the previous literature, most of them have focused mainly on the detection of AFs residues in poultry feeds and there are limited studies about the microbiota of BC rations used in the farms at Sohag Governorate, Egypt, especially AFT *A*. *flavus*. Moreover, very limited research has evaluated antifungal activity (AA) of innovative, eco-friendly nano-particles, such as curcumin nanoparticles (Cur-NPs) and biological agents, such as *S. cerevisiae* against the locally circulating AFT *A. flavus* isolates in Egypt.

Therefore, region-specific, seasonally stratified studies on *A. flavus* AFTG and evaluation of the natural antifungal alternatives are urgently required to support effective preventive strategies and enhance feed and food safety. So, this study aimed to assess fungal contamination of these rations during the different year seasons and to investigate AFTG of *A. flavus* isolates with determination of produced AFB1 level and prevalence of AFs genes (*nor-1* and *ver-1*) among the strong AFT *A. flavus* isolates. AA of Cur-NPs and *S. cerevisiae* against AFT* A. flavus* isolates were further evaluated.

## Material and methods

### Sampling

A total of 240 samples of BC rations were collected randomly from the different farms that had health problems in the flocks at Sohag Governorate, Egypt, across the different seasons of the year (60 samples at each season). The samples were representative, where each sample was a mixture of three samples per farm, including the trough, and it was collected in a sterile polythene bag and transported immediately to the laboratory for mycological analysis [8, 44].

### Mycobiota determination

Total mould count (TMC) was determined on solid media by using the surface-spread technique. 25 g from each sample were mixed with 225 ml of peptone water 0.1% (Oxoid, UK) and homogenized for 20 min. at 2500 rpm. The mixture was left for 2 min. at room temperature, then a ten-fold decimal dilution was prepared from it using peptone water 0.1% [45].

Later, 100 µL from each dilution was spread onto three plates containing dichloran rose bengal chloramphenicol agar (HiMedia Laboratories, India), and then incubated aerobically in the dark at 25 °C for 7–10 days, with daily examination for mould growth. On the last day of the incubation, only the plates containing 10–100 colony-forming units (CFU) were used for counting, and the results were expressed as CFU/g of the sample [46].

Finally, the isolated fungi were sub-cultured on Czapek-yeast extract agar (HiMedia Laboratories, India) and malt extract agar (HiMedia Laboratories, India) by the three-point technique and incubated at 25ºC for 7–14 days in the dark [47]. Identification of fungal genera was carried out according to Pitt and Hocking [48], and Samson et al*.* [49], and *Aspergillus* spp. were identified according to Klich [50].

### Determination of aflatoxigenicity of *A. flavus* isolates

AFT potential of all *A. flavus* isolates was determined by ammonia vapour (AV) assay. The isolates were cultured on coconut agar medium (CAM) and incubated in the dark at 30ºC for 3 days. After incubation, the plates were upended, then 1 drop of 25% ammonia solution (Merck, Germany) was placed into the lid of each plate; distilled water was used as a control negative. Development of pink color is indicative of AFs production, and AFT isolates were classified into four groups according to pink color intensity [51].

### Estimation of AFB1 produced by strong aflatoxigenic *A. flavus* isolates

AFB1 produced by the strong AFT *A. flavus* isolates was semi-quantitatively estimated by thin-layer chromatography (TLC). Each isolate was inoculated into 50 ml of yeast extract (Difco, USA) containing 15% sucrose (Piochem, Egypt) and incubated at 25 °C for 21 days. After the end of the incubation period, 25 ml of chloroform (Piochem, Egypt) was added to each inoculated flask, and the mixture was thoroughly mixed in an ultrasonicator (Hielscher, Germany) for 1 min. Then, it was centrifuged at 3000 rpm for 10 min. The chloroform was decanted, and the extraction by chloroform was repeated. After that, 1 ml of ethanol (EL Nasr, Egypt), 3 g of copper (II) carbonate hydroxide (Piochem, Egypt), and 5–10 g of anhydrous sodium sulphate (EL Nasr, Egypt) were added to the extract, mixed thoroughly, and filtered. Finally, the filtrate was evaporated by warming over a boiling water bath till dryness, then cooled [52].

The residue extracted from each isolate was dissolved in 97:3 of toluene: acetonitrile (Piochem, Egypt), then TLC plates were spotted with the extracted samples at 60 °C, along with standards of 25, 50, 75, and 100 μg/L of AFB1 (Sigma-Aldrich, USA). After that, the plates were placed in a chromatography tank pre-filled with 9:1 of chloroform (Piochem, Egypt): acetone (EL Nasr, Egypt) to about 1 cm from the top of the plate, dried, and dipped into 90:10 of methanol: sulphuric acid (EL Nasr, Egypt). After heating at 150 °C, the plates were examined under UV light at 365 nm, and AFB1 levels were estimated by comparison with the standard [53].

### Genotyping characterization of strong aflatoxigenic *A. flavus* isolates

In this study, 10 randomly selected strong AFT *A. flavus* isolates were genotypically confirmed and identified as *A. flavus* by the detection of the *18S rRNA* gene of *Aspergillus* spp. then sequencing analysis for the fungal Internal Transcribed Spacer (*ITS*) region gene. After that, these isolates were investigated for the presence of two genes involved in the AF biosynthetic pathway: *nor-1* and *ver-1* genes. The used oligonucleotide primers (Metabion, Germany) are listed in Table 1.

**Table 1:**
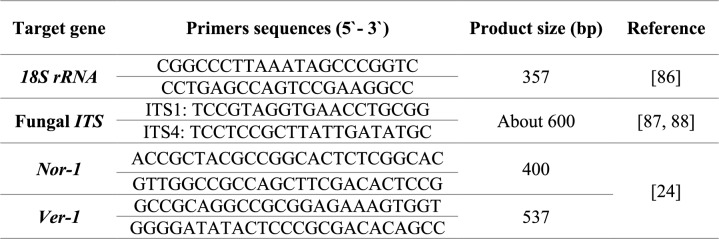
Target genes and oligonucleotide primers used in the study

a Extraction of DNA

Using QIAamp DNeasy plant Mini Kit (Qiagen GmbH, Germany), fungal DNA was extracted from the investigated isolates according to the manufacturer's instructions. After measuring its concentration by NanoDrop 1000 (Thermo Fisher Scientific, USA), the extracted DNA was stored at − 20 ºC till be used.

b Amplification of DNA

The targeted genes were amplified by PCR in a T3 thermocycler (Biometra, Germany) by using EmeraldAmp GT PCR Master Mix (Takara, Japan) and under the conditions illustrated in Table 2 for each target gene. The reaction mixture of PCR was prepared in 50 μl volumes according to the master mix manufacturer's instructions.

**Table 2:**
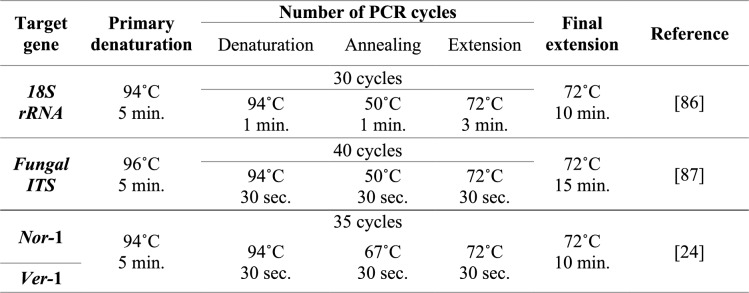
PCR conditions used for each target gene in the study

### Analysis of PCR products

PCR products were investigated by their electrophoresis with 100 bp DNA ladder (Thermo Fisher Scientific, Lithuania) in a 1.5% agarose gel (Biometra, Germany), then photographing of the gel by using gel documentation system (Alpha Innotech, USA) and data analysis.

#### Sequencing of the ITS region gene and phylogenetic analysis

Amplicons of ITS region gene were excised from the agarose gel with a sharp scalpel, then were purified by using the QIAquick gel extraction kit (Qiagen GmbH, Germany) according to the manufacturer's instructions. After that, the purified PCR products were sequenced in an ABI 3500 Genetic Analyzer (Applied Biosystem, USA) by using BigDye™ Terminator v3.1 Cycle Sequencing Kit (Applied Biosystem, USA) according to the manufacturer's instructions.

The obtained sequences of isolates' ITS region gene were compared with those already deposited at the NCBI database by using the BLAST search tool [57] and the CLUSTAL W multiple sequence alignment program, version 1.83 [58]. By using maximum composite-likelihood and neighbor-joining methods in the MEGA program version 6, evolutionary distances were computed, and a phylogenetic tree for the sequences was constructed, respectively [59].

#### Evaluation of antifungal activity of curcumin nanoparticles and Saccharomyces cerevisiae against aflatoxigenic *A. flavus* isolates

Each AFT *A. flavus* isolate was grown on potato dextrose agar (HiMedia Laboratories, India) at 30ºC for 7 days, and a homogenous spore suspension was obtained in 10 ml normal saline containing 0.1% tween 80 (EL Nasr Company, Egypt), then it was adjusted to 10^6^ spore/ml by using a hemocytometer [41].

The freshly prepared fungal suspension was swabbed evenly on the entire surface of Sabouraud's dextrose agar (SDA) (HiMedia Laboratories, India) and left for 5 min. to dry. After that, by using a sterile cork borer, 5 wells of 6 mm diameter were punched on the agar surface, 1 in the agar center and 4 peripherals to it, and at equal distances. Each well was then filled with 100 μl of 0.5, 1, 2, and 4 mg/ml of Cur-NPs, while the central well was filled with 100 μl of distilled water as a negative control, and the inoculated plates were incubated at 30ºC for 5 days. Antifungal efficiency of Cur-NPs was assessed by measuring the growth inhibition zone diameters (mm) formed around the wells [41]. Cur-NPs were purchased from Nanotech, Egypt, and their characteristics included appearance “yellowish-brown powder”, solubility “suspension in water and colloidal in Ethanol”, shape (TEM) “spherical-like shape”, size (TEM) “50 ± 5.5 nm”, and optical properties (Abs.) “λmax = 425 nm”.

While AA of *S. cerevisiae* was determined by swabbing the freshly prepared fungal suspension of each AFT *A. flavus* isolate evenly on the entire surface of SDA and left it for 5 min. to dry. After that, by using a sterile cork borer, 2 wells of 6 mm diameter were punched on the agar surface. One well was then filled with 100 μl of *S. cerevisiae* (10^6^ cells/ml) while the other well was filled with 100 μl of distilled water as a negative control, and the inoculated plates were incubated at 30ºC for 5 days. Antifungal efficiency of *S. cerevisiae* was assessed by measuring the growth inhibition zone diameters (mm) formed around the wells [60].

### Statistical analysis

In the current study, data generated were presented as percentages, as well as the results of total mould count, produced AFB1 level by the strong AFT *A. flavus* isolates, and AA of Cur-NPs and *S. cerevisiae* against AFT *A. flavus* isolates were expressed as means ± SE. Data was statistically analyzed using IBM SPSS software (version 22.0), and the incidence of fungal contamination, different fungi genera*, Aspergillus* spp., as well as aflatoxigenic *A. flavus* isolates among the examined rations through the different year seasons tested using chi-square. A *P*-value less than 0.05 was considered significant.

## Results

### Mycobiota of the examined rations

Mycological examination revealed that 92.9% of the examined rations were contaminated by fungi (Table 3), and TMC ranged from 1 × 10 to 1.91 × 10^7^ CFU/g of ration, with an average of 1.18 × 10^5^ (± 8.78 × 10^4^) CFU/g of ration (Table 4). Table 5 illustrates the frequency distribution of TMC in the examined rations during the different year seasons.

**Table 3:**
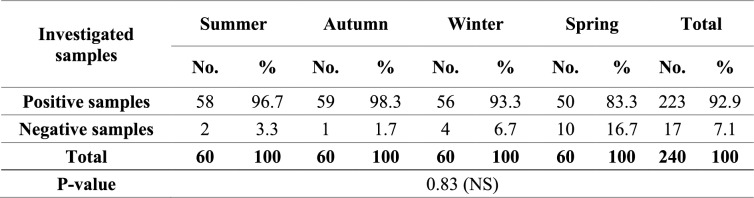
Incidence of contamination with fungi among the examined rations during the different year seasons

**Table 4:**
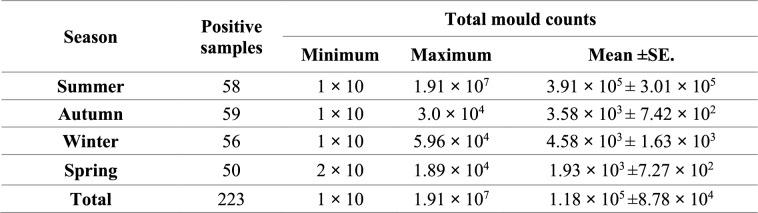
Total mould counts in the examined rations during the different year seasons

**Table 5:**
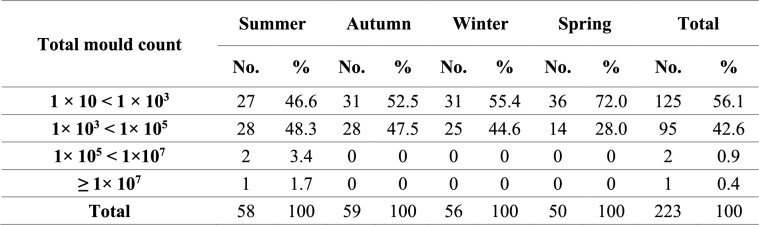
Frequency distribution of total mould counts in the examined rations during the different year seasons

Altogether, fungal isolates belonging to 27 fungal genera were recovered from the examined ration samples as illustrated in Table 6. *Aspergillus* was the most predominant genus, followed by *Penicillium*, *Mucor*, *Cladosporium*, *Eurotium,* and *Scopulariopsis,* while the other fungal genera were recovered with small frequency. Furthermore, *Aspergillus* isolates were characterized morphologically into 20 different species with predominance of *A. flavus*, *Aspergillus niger* (*A. niger*), *Aspergillus terreus* (*A. terreus*)*,* and *Aspergillus ustus* (*A. ustus*)*,* respectively, as illustrated in Table 7 and Figures S1-S9.

**Table 6:**
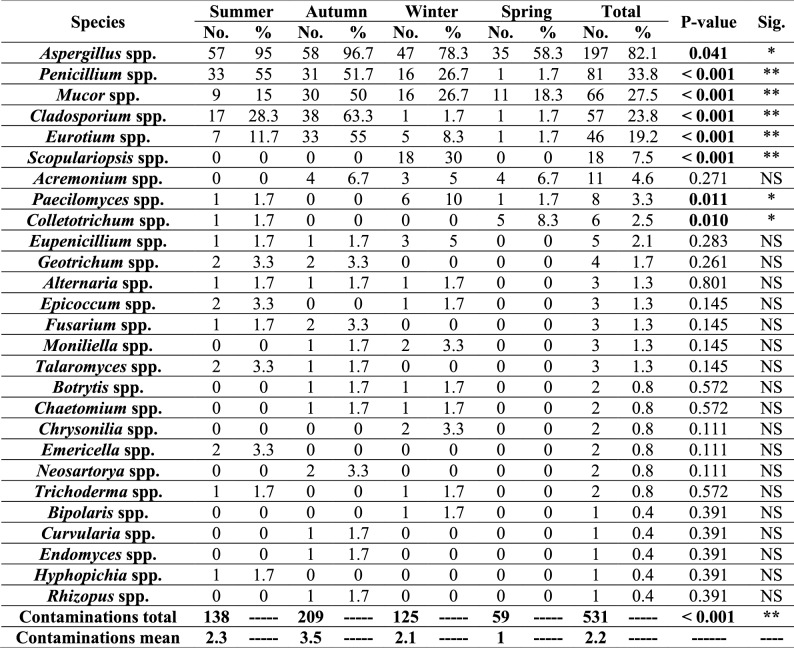
Incidence of different fungi genera among the examined rations during the different year seasons

**Table 7:**
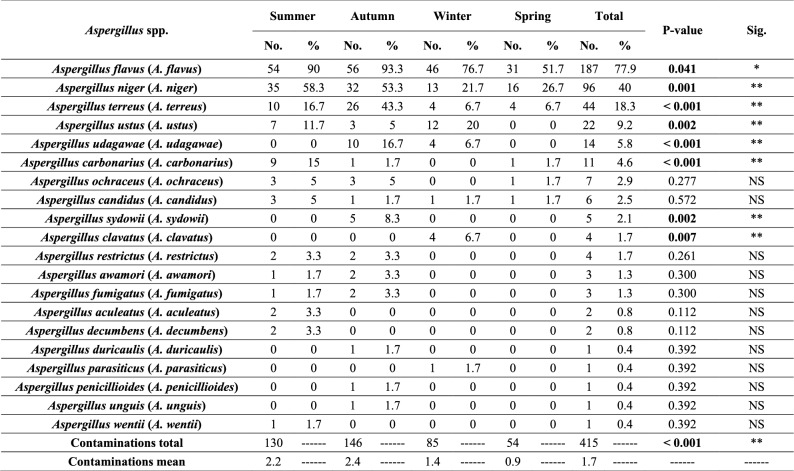
Incidence of Aspergillus spp. among the examined rations during the different year seasons

With respect to the seasonal variation in the contamination with fungi, data illustrated in Tables 3, 6, and 7 showed that contamination of the investigated rations with fungi was the highest in autumn, followed by summer, winter, and spring, respectively, and statistical analysis revealed that there were significant differences in the prevalence of different fungal genera and *Aspergillus* spp. between the different year seasons.

### Aflatoxigenicity of *A. flavus* isolates

Investigation of *A. flavus* isolates by AV assay revealed that 27.3% of them were AFT with variable degrees, as illustrated in Table 8 and Figure S10. Most AFT *A. flavus* was isolated from the ration samples collected during the autumn and summer seasons, and statistical analysis revealed that there were significant differences in the prevalence of AFT *A. flavus* between the different year seasons.

**Table 8:**
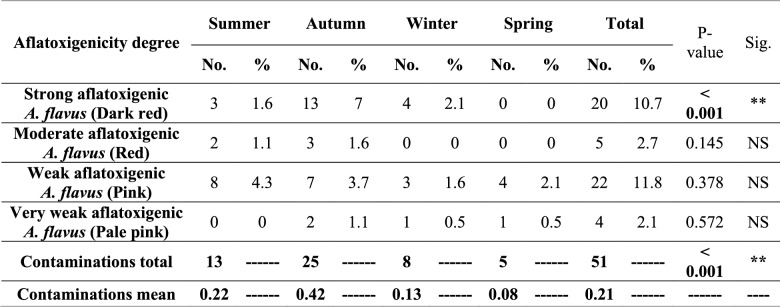
Distribution of aflatoxigenic A. flavus isolates through the different year seasons

### AFB1 production by strong aflatoxigenic *A. flavus *isolates

Investigation of strong AFT *A. flavus* isolates by TLC for AFB1 production revealed that 90% of these isolates produced AFB1 with high amounts ranging from less than 25 to 71 µg/L. Table 9 illustrates AFB1 produced by the investigated isolates through the different year seasons.

**Table 9:**
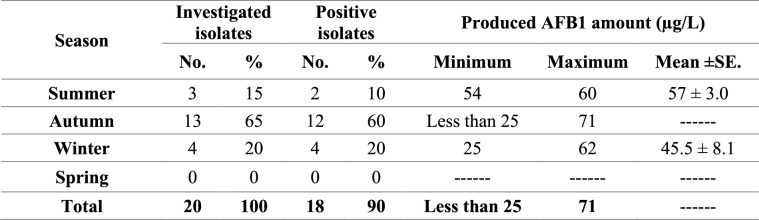
AFB1 produced by strong aflatoxigenic A. flavus isolates during the different year seasons

### Genotyping characterization of strong aflatoxigenic *A. flavus* isolates

**Fig. 1:**
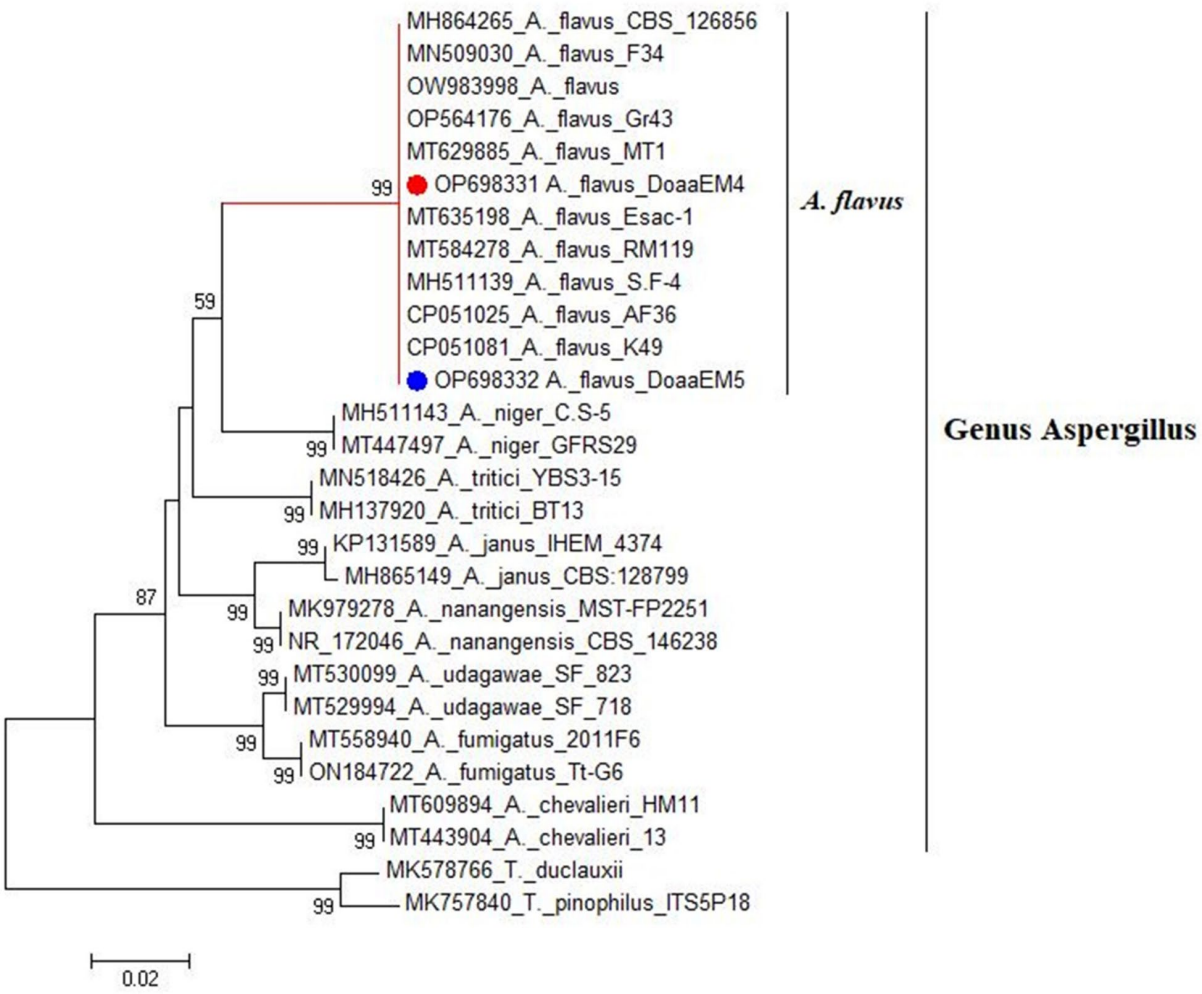
Phylogenetic tree showing the evolutionary relationship of *A. flavus* DoaaEM4 and DoaaEM5 isolates with the other *Aspergillus* spp., based on the ITS region gene sequences evolutionary distance

**Fig. 2:**
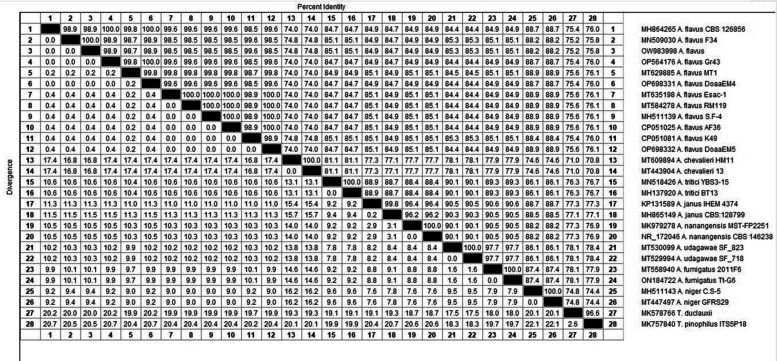
Identity and divergence percent between *A. flavus* isolates of this study and *Aspergillus* spp. stored in GenBank database based on ITS region gene sequences

Genotypically, the investigated isolates by PCR (*n* = 10) were confirmed as *A. flavus* by detection of *Aspergillus 18S rRNA* gene, as illustrated in Figure S11, as well as sequencing analysis for fungal ITS region gene (Figure S12). Comparison of the ITS region gene sequences of our *A. flavus* isolates with the ITS region gene sequences of *Aspergillus* spp. stored in the GenBank database, revealed homology of 98.5 to 100% with stored *A. flavus* sequences compared with homology of 74.0 to 88.9% with the stored sequences of the other *Aspergillus* spp., as illustrated in Figs. 1 and 2. ITS region gene sequences of our *A. flavus* isolates DoaaEM4 and DoaaEM5 were deposited into NCBI and assigned accession numbers OP698331.1 and OP698332.1. Seven isolates shared an identical sequence corresponding to accession number OP698331.1, while the remaining three isolates exhibited an identical sequence corresponding to accession number OP698332.1.

In terms of harboring the investigated strong AFT and AFB1 producer* A*. *flavus* isolates for AFs genes, PCR showed the presence of *nor*−1 gene in all the investigated isolates, while *ver*−1 gene was present in only 30% of them, as illustrated in Figures S13 and S14, respectively, and Table 10.

**Table 10:** Incidence of *nor*−1 and *ver*−1 genes among the investigated aflatoxigenic *A*. *flavus* isolates

Investigated isolate number	Amount of produced AFB1 (µg/L)	Nor-1 gene	Ver-1 gene
1	70	+	+
2	50	+	-
3	54	+	-
4	54	+	-
5	25	+	-
6	62	+	+
7	60	+	+
8	45	+	-
9	71	+	-
10	25	+	-
Total (%)		100	30

### Antifungal activity of curcumin nanoparticles and Saccharomyces cerevisiae against aflatoxigenic *A. flavus* isolates

As illustrated in Table 11, Cur-NPs exhibited strong AA against AFT *A. flavus* isolates, and their AA increased with increasing concentration (Figure S15). Also, the growth of all the AFT *A. flavus* isolates was inhibited with *S. cerevisiae* (10^6^ cells/ml) with an inhibition zone of 24.6 ± 0.64 mm^2^ (Figure S16).

**Table 11:** Antifungal activity of different curcumin nanoparticle concentrations against aflatoxigenic *A. flavus* isolates

	**Concentration of curcumin nanoparticles (mg/ml)**			
	**0.5**	**1**	**2**	**4**
Inhibition zone (mm2)	11.2 ± 0.69	13.7 ± 0.62	16.4 ± 0.64	19.2 ± 0.65
Inhibition rate for aflatoxigenic A. flavus isolates (%)	88.2	98	100	100

## Discussion

In addition to having an impact on feed quality and its organoleptic qualities, feed contamination with pathogenic fungi and MTs constitutes a serious hazard to both humans and animals [61], particularly in the tropical and subtropical regions [13]. This study provided significant data about the magnitude of mycobiota in BC rations used in farms at Sohag Governorate, Egypt, throughout the various seasons, particularly *Aspergillus* spp. and AFTG of *A. flavus*, in addition to AA of Cur-NPs and *S. cerevisiae* against *A. flavus* isolates. It is anticipated that these findings will help BC rations enterprises, farmers, and investors to regulate and/or prevent fungal and MTs contamination.

TMC is a helpful indicator to determine the hygienic quality of feed; it shouldn't exceed the value of 1 × 10^5^ CFU/g [62]. As illustrated in Tables 3 and 4, 92.9% of the examined rations in this study were contaminated by the fungi with TMC ranging from 1 × 10 to 1.91 × 10^7^ CFU/g of ration and exceeding the maximal allowable limit in only 1.3% of the examined ration samples, as illustrated in Table 5. Our findings concurred with those of Ibrahim et al*.* [63], who reported that fungi contaminated 94.33% of poultry feed samples, whereas Osho et al*.* [64] and Sultana et al*.* [65] found that fungi contaminated 100% and 10% of poultry feed samples, respectively. Additionally, we found that TMC in chicken feed samples was nearly below 1 × 10^5^ CFU/g of ration, but Rosa et al*.* [66] found it to be over 1 × 10^5^ CFU/g of ration. Our findings were in close agreement with those of Dalcero et al*.* [62] and Magnoli et al*.* [67].

On the other hand, and as presented in Table 6, it was found that the examined ration samples were contaminated with different fungal genera.* Aspergillus* was the most prevalent genus among the examined samples, followed by *Penicillium*, *Mucor*, *Cladosporium*, *Eurotium*, and *Scopulariopsis*, respectively, while the other fungal genera were recovered with small frequency. These results agreed with those of Saleemi et al*.* [68], who reported that *Aspergillus* spp. were the predominant among poultry feeds, followed by *Penicillium* spp. while Osho et al*.* [64] reported that *Rhizopus* spp. were the predominant among poultry feeds, followed by *Fusarium* spp. and *Aspergillus* spp., respectively. Dominance of *Aspergillus* spp. in this study may be attributed to their widespread occurrence in the environment [69].

Furthermore, *Aspergillus* isolates in this study were morphologically characterized, as illustrated in Table 7. *A. flavus* was the most prevalent species among the examined samples, followed by *A. niger*, *A. terreus*, and *A. ustus*, respectively, in agreement with Azarakhsh et al. [4], Hassan et al. [8], and Osho et al. [64], who reported that *A. flavus* followed by *A. niger* were the most prevalent species among poultry feeds, while Habib et al. [6] demonstrated that *Aspergillus fumigatus* and *Aspergillus parasiticus* (*A. parasiticus*) were the most prevalent species among poultry feeds in Kaduna State, Nigeria. The differences in the fungal contamination between our study and the other studies could be attributed to the differences in the geographical location, the year [70], atmospheric conditions, feed storage conditions, and feed ingredients [71], feed storage period, and feed moisture contents [19]**.**

Although the examined rations in this study were ready-to-serve and within shelf life, their fungal contamination was very high, and this may be attributed to the unhygienic conditions during harvesting of their ingredients, storage, processing, production, and consumption [70], as well as their exposure to high temperatures and humidity during these stages [32]**,** which is supported by their higher fungal contamination during autumn and summer seasons, where these conditions are conducive to fungal growth [5]. This situation requires performing the necessary control measures immediately, maintaining optimum feed production, processing, and storage, as well as periodically checking the mycological state of the feeds to maintain human health, conserve food quality, and maintain BC's health and productivity.

AFs are the most common and toxic MTs of concern in poultry feeds [8]. They are mainly produced by *A. flavus* and *A. parasiticus* [18]. AF-producing potential of *A. flavus* isolates is highly variable, ranging from strong AF producers to non-AFT isolates [51]. Culture technique has been widely used for the identification of AFTG of *A. flavus* isolates [15]. According to Fani et al. [51], CAM in combination with AV is a rapid, cheap, semi-quantitative AF assay, and its results were in total agreement with those from HPLC and TLC. 27.3% of *A. flavus* isolates in this study were AFT with variable degrees, in agreement with Nurtjahja et al. [15] and Sana et al. [72], who found that 28.1% and 29.8% of *A. flavus* isolates were AFT, respectively, while Alkhursan et al. [11], Faparusi and Alagamba [73], and Motbaynor et al. [19] reported that 52%, 16.1%, and 73.2% of *A. flavus* isolates from poultry feeds were AFT, respectively.

AFB1 is the most prevalent and dangerous AF [19]. Several methods are available for AFs determination [72]. TLC is one of the most widely used techniques for AFs determination [74], and is a more precise method for AFB1 determination [44]. In the present study, all the strong AFT *A*. *flavus* isolates were investigated by TLC for AFB1 production, and the results revealed that 90% of them were AFB1 producers, with high amounts ranging from less than 25 to 71 µg/L. Similar findings were reported by Fakruddin et al. [75], who found that 93.3% of *A*. *flavus* isolates were AFB1 producers, while Iram et al. [5] and Nurtjahja et al. [15] reported that 100% and 64.3% of *A. flavus* isolates were AFB1 producers, respectively, and this variation, as well as the variations in *A. flavus* AFTG, could be attributed to the differences in the geographical location [76], genetic differences [69], and atmospheric and feed storage conditions [71]. On the other hand, most AFT *A. flavus* isolates and AFB1-producers were reported in autumn, followed by summer, and this could be attributed to the environmental conditions [76].

High prevalence of AFT *A. flavus* and AFB1-producers reported in this study alarms us about the high potential of BC rations contamination with AFs and AFB1, posing threats to both BC and humans; therefore, immediate necessary control measures are demanded. Also, since the application of non-AFT *A*. *flavus* isolates is a highly effective strategy for natural AFs mitigation through the competitive exclusion [51], our native characterized non-AFT *A*. *flavus* isolates will be further investigated and tested for their potential as biological control agents for AFs mitigation in BC rations.

Identification of fungi based on the morphological characteristics has been supported by many studies; however, the convergent evolution of fungi has resulted in the inaccuracy of the traditional methods for the identification of fungi in some cases [77]. Molecular methods complementing the morphological ones are very promising for identifying fungal species [78]. Therefore, in this study, the investigated isolates for AFs genes were genotypically confirmed as *A. flavus* by detection of the *Aspergillus 18S rRNA* gene, which is a valuable taxonomic tool for *Aspergillus* section *Flavi* [79] and sequencing analysis of the ITS region gene, which is a standard molecular marker that has been used for the identification of most fungal species [78].

There is a genetic diversity in the biosynthesis pathway of AFs among *A. flavus* isolates. This pathway contains 23 enzymes encoded by 25 genes clustered in a 70-kb DNA region on chromosome III [24]. In this study, 10 randomly selected strong AFT *A. flavus* isolates were investigated for the presence of *nor-1* and *ver-1* genes. The results showed the presence of the *nor-1* gene in all the investigated isolates, in agreement with Nurtjahja et al. [15] and in exact correlation with the determination results of AFTG by AV assay and AFB1 production by TLC, as reported by Ronoh et al. [80] and Mohamed et al. [77], respectively. With respect to *the ver-1* gene, only 30% of the investigated isolates harbored the *ver-1* gene, while Gherbawy et al. [76] reported that 71.4% of *A. flavus* isolated from poultry feedstuff samples harbored the *ver-1* gene, and this difference could be attributed to the geographical location. On the other hand, our results agreed with those of Fakruddin et al. [75], who found that some AFT *A. flavus* isolates didn’t harbor the *ver-1* gene, and this discrepancy may be attributed to the fact that the used primers were unable to amplify the *ver-1* gene in these isolates [75].

Curcumin is a significant bioactive ingredient present in the rhizomes of the medicinal plant, *Curcuma longa*. In recent years, it has gained immense attention due to the variety of its biological and pharmacological activities: anti-inflammatory, anti-oxidant, anti-tumor, and antimicrobial activity [81]. Many studies have reported the effectiveness of Cur-NPs against different microorganisms at a very low concentration [82]. In this study, Cur-NPs exhibited strong AA against AFT *A. flavus* isolated from BC rations, so it is a promising candidate to be used as a natural, safe, and cheap preservative to control AFT *A. flavus* and AFs in these rations. Similar to our results, Alnashi and Abdel Fattah [83] and Sehim et al. [41] recorded that the different concentrations of Cur-NPs exhibited strong AA against *A. flavus* isolates, and the latter attributed the reported AA to ergosterol biosynthesis inhibition, PM-ATPase inhibition, and proteinase secretion.

*S. cerevisiae* has been known as a safe, non-pathogenic yeast [84] and widely used for thousands of years by humans [85]. *S. cerevisiae* strains showed variable AA against several fungi [86]. In this study, *S. cerevisiae* inhibited the growth of all AFT *A. flavus* isolates in agreement with the results of Al-Aedany and Mohammad [87] and Oluwole et al. [60], and this suppression may be attributed to the competition for nutrients and space, the direct contact, and hydrolytic enzyme production [34, 88]. The broader implications of our result include that *S. cerevisiae* could be a promising, safe, cheap, and eco-friendly biological agent to control AFT *A. flavus* and AFs in BC feeds, and this method may provide additional probiotic effects after ingestion of the treated feeds in addition to adsorption of MTs [43].

## Conclusion

According to this study, the majority of the ready-to-serve rations in BC farms at Sohag Governorate, Egypt, were contaminated by various fungal species, particularly *Aspergillus*, *Penicillium*, *Mucor*, and *Cladosporium* species. Additionally, there was a notable prevalence of AFT *A. flavus* among these rations, which poses a risk to both BC and humans, and the associated financial losses. As a result, immediate essential control and preventive measures must be taken to assure the safety of feed and food. Cur-NPs and *S. cerevisiae*, on the other hand, demonstrated strong AA against AFT *A. flavus* isolates. As such, it is advised that they be evaluated and used as safe, affordable, and environmentally friendly fungicides to control AFT *A. flavus* and its potential to produce AFs in BC feeds.

## Supplementary Information

Supplementary Material 1.

## Data Availability

The datasets generated and/or analyzed during the current study are available in the GenBank database under accession numbers OP698331.1 (https://www.ncbi.nlm.nih.gov/nuccore/OP698331.1/) and OP698332.1 (https://www.ncbi.nlm.nih.gov/nuccore/OP698332.1).

## Abbreviations

*A. flavus*: *Aspergillus flavus*
*A. niger*: *Aspergillus niger*
*A. parasiticus*: *Aspergillus parasiticus*
*A. terreus*: *Aspergillus terreus*
*A. ustus*: *Aspergillus ustus*
AA: Antifungal activity
AFB1: Aflatoxin B1
AFs: Aflatoxins
AFT: Aflatoxigenic
AFTG: Aflatoxigenicity
AV: Ammonia Vapour
BC: Broiler chickens
CAM: Coconut agar medium
CFU: Colony forming units
Cur-NPs: Curcumin nanoparticles
ITS: Internal Transcribed Spacer
MTs: Mycotoxins
*S. cerevisiae*: *Saccharomyces cerevisiae*
SDA: Sabouraud's dextrose agar
TLC: Thin layer chromatography
TMC: Total mould count
