# Supplementary material for: Antifungal activity of curcumin nanoparticles and Saccharomyces cerevisiae against aflatoxigenic Aspergillus flavus isolated from broiler chickens rations in Egypt
Source: BMC Microbiol. 2026 Apr 29;26:421. doi: 10.1186/s12866-026-04963-3 (PMC13130673; doi:10.1186/s12866-026-04963-3)
Supplement: Supplementary file 1 — Supplementary Material 1. [file 12866_2026_4963_MOESM1_ESM.docx]

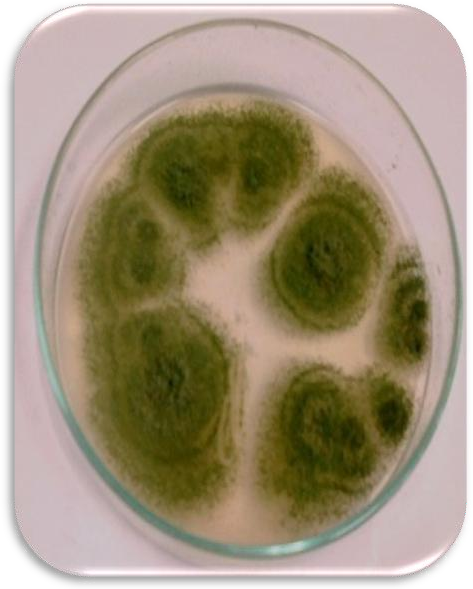

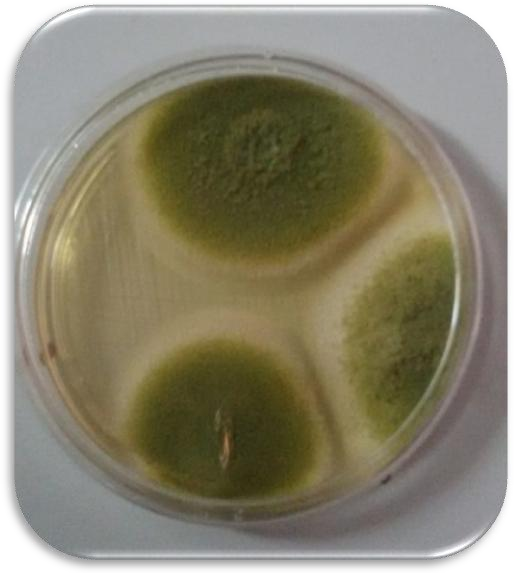


**Figure S1.** 7-day-old cultures of *A. flavus* on malt extract agar (left) and Czapek-yeast extract agar (right) (at 25ºC).


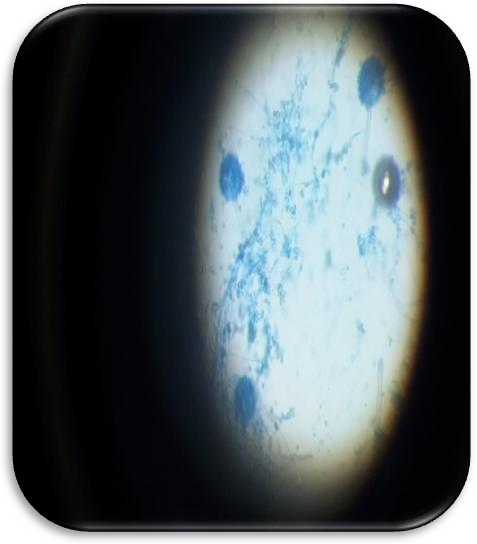

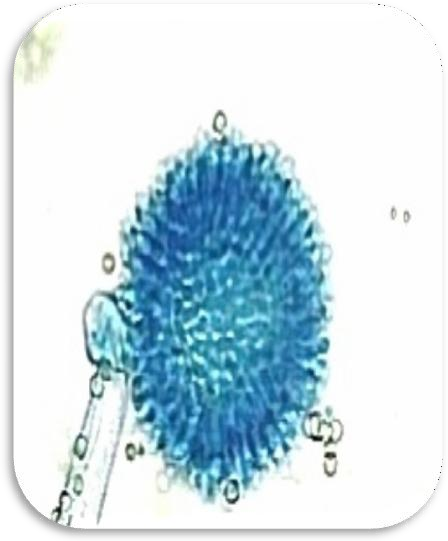


**Figure S2.** Microscopical appearance of *A. flavus* stained with lactophenol cotton blue (left by low power and right by high power).


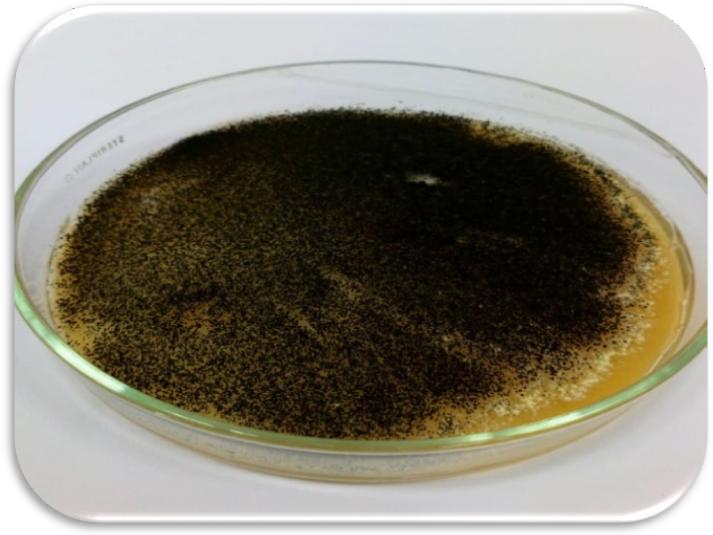


**Figure S3.** 7-day-old culture of *A. niger* on malt extract agar (at 25ºC).


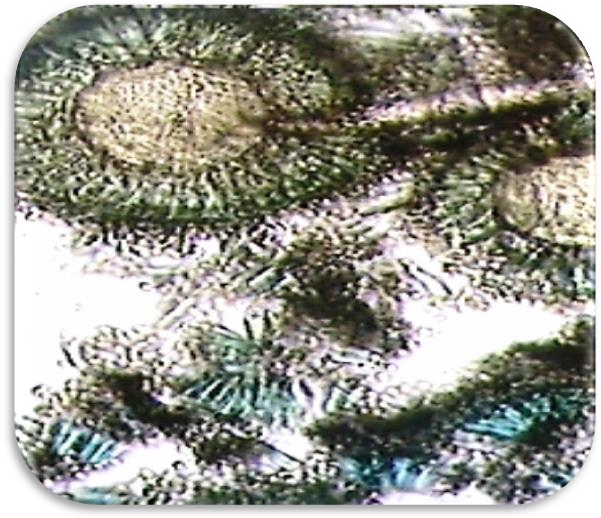


**Figure S4.** Microscopical appearance of *A. niger* stained with lactophenol cotton blue (by high power).


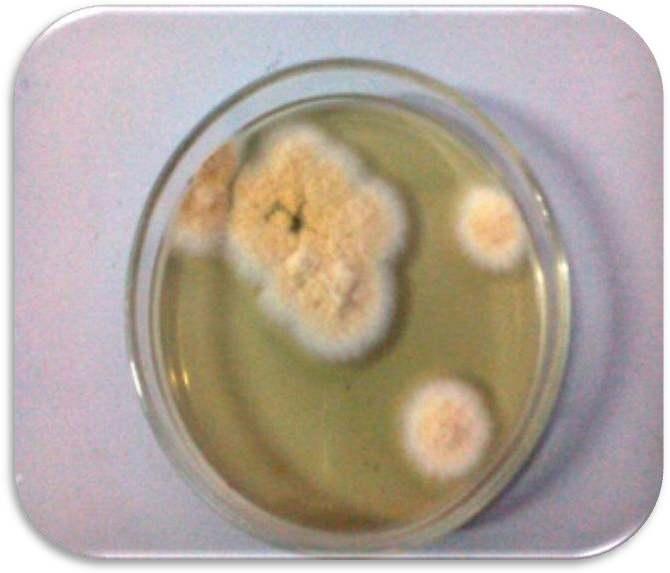


**Figure S5.** 7-day-old culture of *A. terreus* on malt extract agar (at 25ºC).


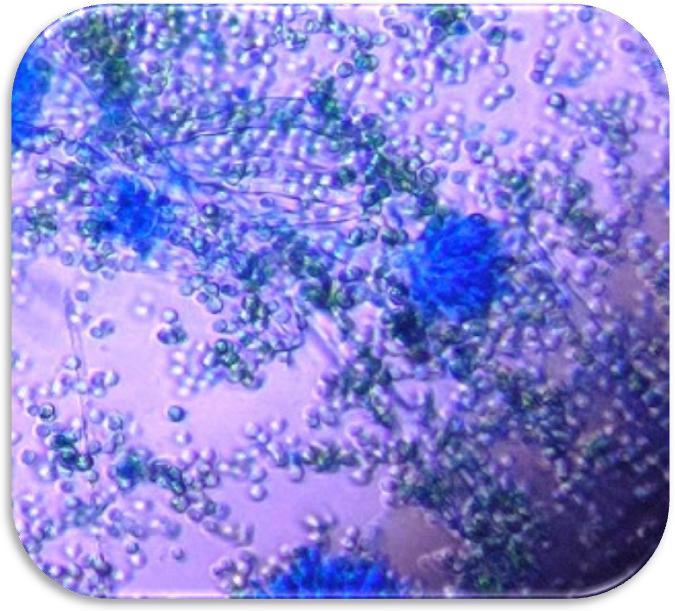


**Figure S6.** Microscopical appearance of *A. udagawae* stained with lactophenol cotton blue (by high power).

**
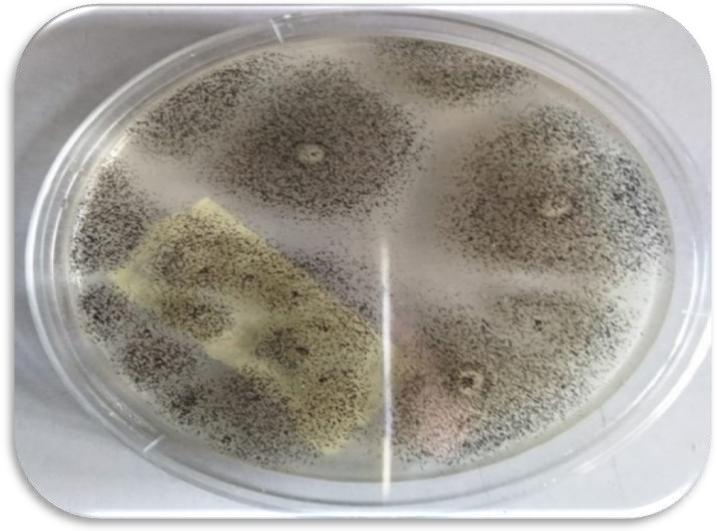
Figure S7.** 7-day-old culture of *A. carbonrius* on malt extract agar (at 25ºC).


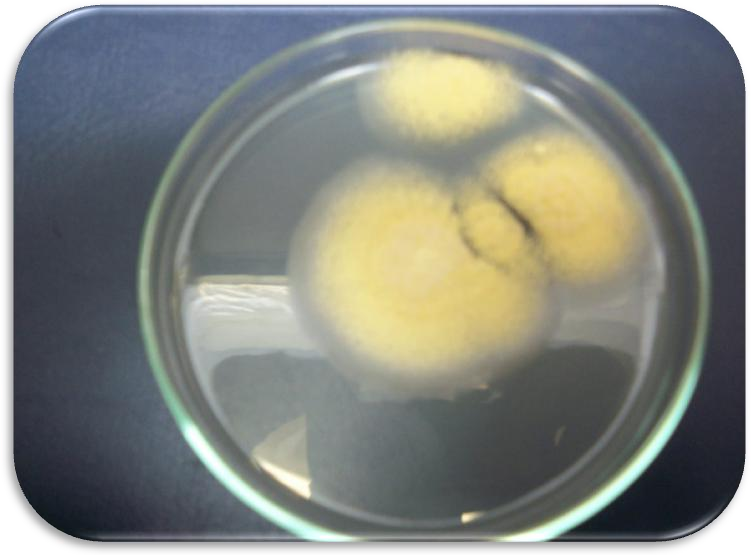


**Figure S8.** 7-day-old culture of *A. ochraceus* on malt extract agar (at 25ºC).


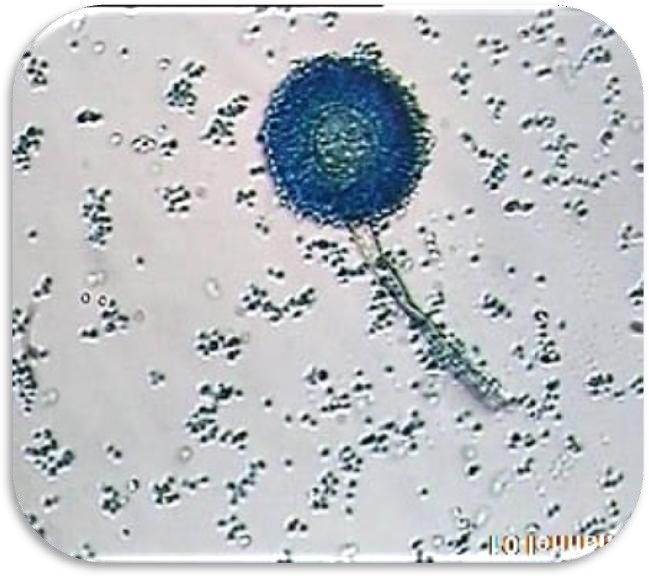


**Figure S9.** Microscopical appearance of *A. ochraceus* stained with lactophenol cotton blue (by high power).


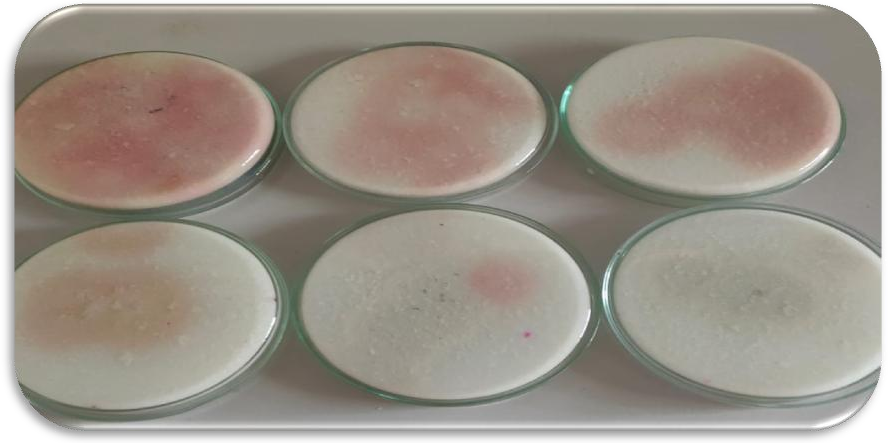


**Figure S10.** Results of aflatoxigenicity determination for *A. flavus* isolates by ammonia vapor assay on coconut agar media. Intensity of the developed pink color differs according to the aflatoxigenicity degree of the *A. flavus* isolate.


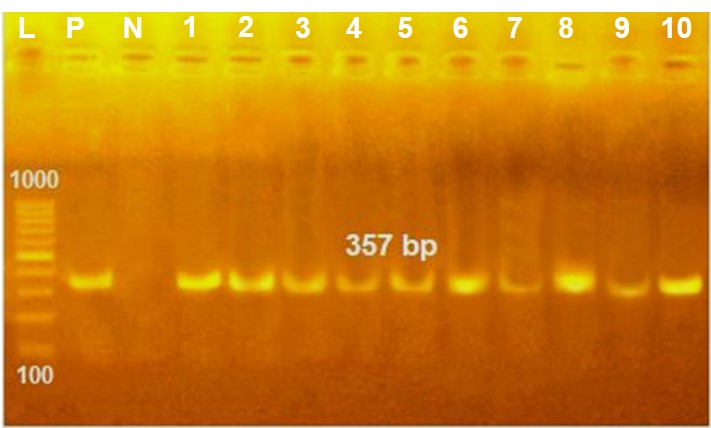


**Figure S11.** Agarose gel electrophoresis for products of PCR, which targeted *18S rRNA* gene in *A. flavus* isolates. Lane L: DNA ladder, Lane P: Positive control, Lane N: Negative control, and Lanes 1-10: PCR products of *A. flavus* isolates showing positive bands at 357 bp in all the investigated isolates.


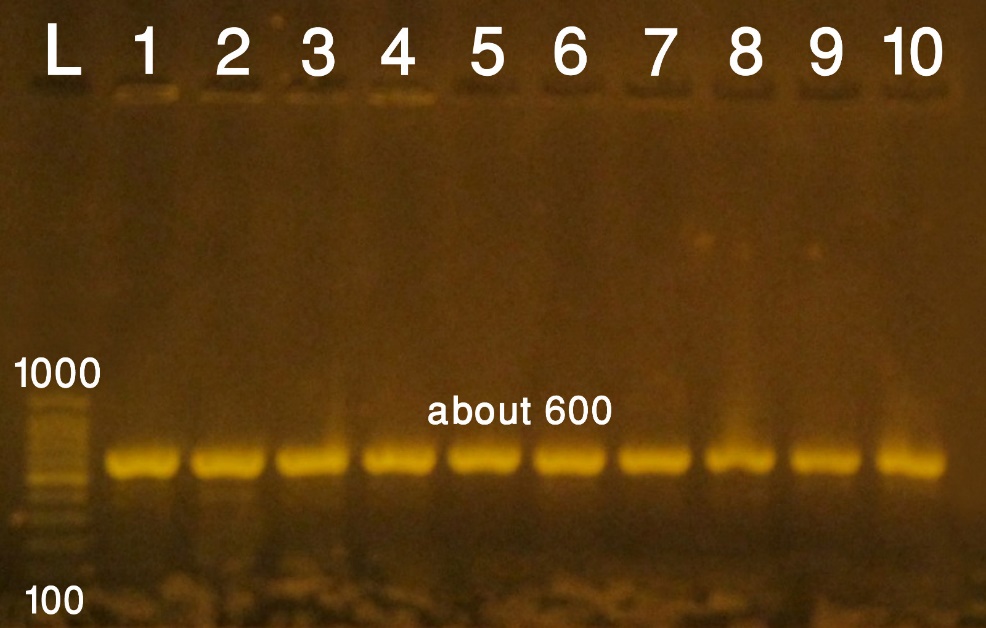


**Figure S12.** Agarose gel electrophoresis for products of PCR, which targeted the ITS region gene in *A. flavus* isolates. Lane L: DNA ladder and Lanes 1-10: PCR products of *A. flavus* isolates showing positive bands at about 600 bp in all the investigated isolates.


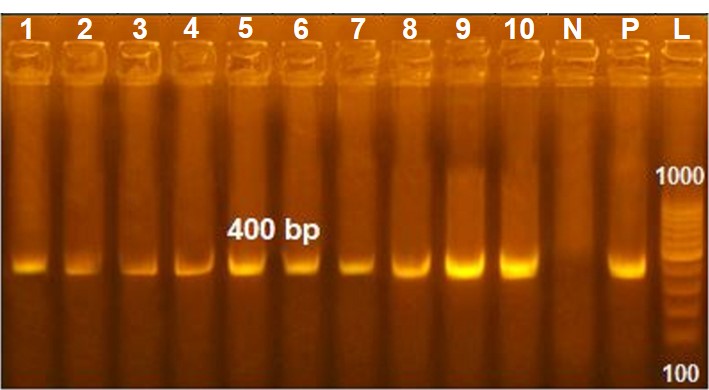


**Figure S13.** Agarose gel electrophoresis for products of PCR, which targeted *nor*-1 gene in *A. flavus* isolates. Lane L: DNA ladder, Lane P: Positive control, Lane N: Negative control, and Lanes 1-10: PCR products of *A. flavus* isolates showing positive bands at 400 bp in all the investigated isolates.


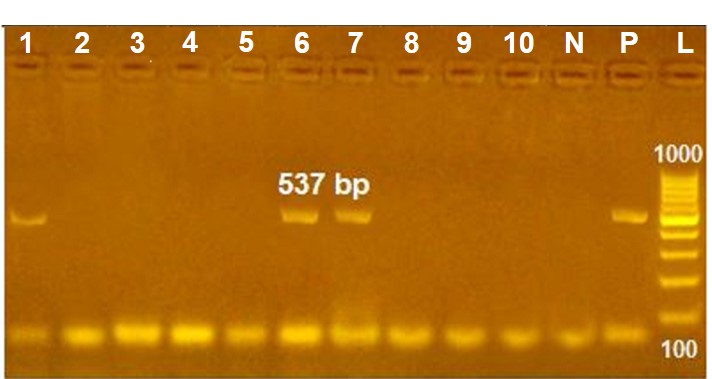


**Figure S14.** Agarose gel electrophoresis for products of PCR, which targeted *ver*-1 gene in *A. flavus* isolates. Lane L: DNA ladder, Lane P: Positive control, Lane N: Negative control, and Lanes 1-10: PCR products of *A. flavus* isolates showing positive bands at 537 bp in isolates number 1, 6, and 7.

**
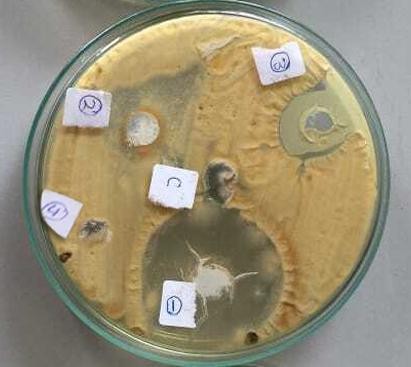
**

**Figure S15.** Antifungal activity of curcumin nanoparticles against aflatoxigenic *A*. *flavus* isolate. C: Well inoculated with 100 μl of distilled water (negative control) while 1, 2, 3 and 4: Wells inoculated with 100 μl of 4, 2, 1 and 0.5 mg/ml of Cur-NPs, respectively.


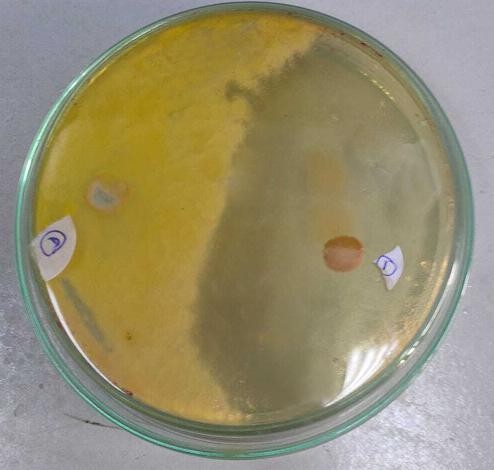


**Figure S16.** Antifungal activity of *Saccharomyces cerevisiae* against aflatoxigenic *A*. *flavus* isolate. C: Well inoculated with 100 μl of distilled water (negative control) and 1: Well inoculated with 100 μl of *Saccharomyces cerevisiae* (10^6^ cells/ml).
